# Language in autism: domains, profiles and co-occurring conditions

**DOI:** 10.1007/s00702-023-02592-y

**Published:** 2023-03-16

**Authors:** Jeannette Schaeffer, Muna Abd El-Raziq, Elena Castroviejo, Stephanie Durrleman, Sandrine Ferré, Ileana Grama, Petra Hendriks, Mikhail Kissine, Marta Manenti, Theodoros Marinis, Natalia Meir, Rama Novogrodsky, Alexandra Perovic, Francesca Panzeri, Silvia Silleresi, Nufar Sukenik, Agustín Vicente, Racha Zebib, Philippe Prévost, Laurice Tuller

**Affiliations:** 1 Department of Literary and Cultural Analysis & Linguistics, Faculty of Humanities, University of Amsterdam, PO Box 1642, 1000 BP Amsterdam, The Netherlands; 2 Bar-Ilan University, Ramat Gan, Israel; 3 University of the Basque Country, Vitoria-Gasteiz, Spain; 4 University of Fribourg, Fribourg, Switzerland; 5 UMR 1253 iBrain, Université de Tours, INSERM, Tours, France; 6 University of Groningen, Groningen, The Netherlands; 7 Université Libre de Bruxelles, Brussels, Belgium; 8 University College London, London, UK; 9 University of Milano-Bicocca, Milan, Italy; 10 University of Haifa, Haifa, Israel; 11 University of Konstanz, Konstanz, Germany; 12 Basque Foundation for Science, Ikerbasque, Bilbao, Spain

**Keywords:** Autism, Language, Language impairment, Comorbidity, Dimensional approach, Neurodevelopmental disorders

## Abstract

This article reviews the current knowledge state on pragmatic and structural language abilities in autism and their potential relation to extralinguistic abilities and autistic traits. The focus is on questions regarding autism language profiles with varying degrees of (selective) impairment and with respect to potential comorbidity of autism and language impairment: Is language impairment in autism the co-occurrence of two distinct conditions (comorbidity), a consequence of autism itself (no comorbidity), or one possible combination from a series of neurodevelopmental properties (dimensional approach)? As for language profiles in autism, three main groups are identified, namely, (i) verbal autistic individuals without structural language impairment, (ii) verbal autistic individuals with structural language impairment, and (iii) minimally verbal autistic individuals. However, this tripartite distinction hides enormous linguistic heterogeneity. Regarding the nature of language impairment in autism, there is currently no model of how language difficulties may interact with autism characteristics and with various extralinguistic cognitive abilities. Building such a model requires carefully designed explorations that address specific aspects of language and extralinguistic cognition. This should lead to a fundamental increase in our understanding of language impairment in autism, thereby paving the way for a substantial contribution to the question of how to best characterize neurodevelopmental disorders.

## Introduction

Autism spectrum disorder (ASD) is a neurodevelopmental condition^1^ whose diagnosis is bi-dimensional, based on the presence of (1) qualitative and significant impairment in socio-communicative abilities and social interaction, as well as (2) restrictive, repetitive, and inflexible behavioral patterns, activities or interests. Deficits must cause clinically significant impairment in the individual's social, academic, occupational, and daily functional areas (APA 2013; WHO 2022). Studies published in recent years emphasize the vast heterogeneity in the abilities of individuals with ASD across different domains of cognitive, social, and linguistic functioning (Masi et al. 2017; Pelphrey et al. 2011).

In current versions of the two principal nosographic systems, the DSM-5 (APA 2013) and the ICD-11 (WHO 2022), language deficits are no longer included in the diagnostic criteria. However, both classifications require specification of accompanying language impairment, just as they require specification of other accompanying conditions (notably, intellectual impairment). The number one reason in most countries around the world that drives parents to seek a formal evaluation and diagnosis is a delayed communication and language onset or poor linguistic abilities compared to age-matched peers (Kozlowski et al. 2011), indicating that a large proportion of children with ASD struggle with language. Language abilities are consistently found to be the most stable predictors of social (Chow et al. 2021; Gonzales et al. 2010) and educational (McKernan and Kim 2022; Spiegel et al. 2021) well-being and success; thus a focus on supporting the language needs is crucial in formulating intervention aims for children with ASD. However, in many clinical settings, language skills of children with ASD are not always assessed, or, even if they are assessed, subtle distinctions between different linguistic abilities are missed out. Studies have reported diverse language profiles in ASD, with some children found to have intact structural language skills (sound structure and grammar) and others displaying language impairments which are at least phenotypically similar to those reported for Developmental Language Disorder (DLD), a neurodevelopmental disorder characterized by persistent deficits in the acquisition, understanding, production or use of language that cause significant limitations in the ability to communicate (WHO 2022). Language abilities in DLD are markedly below age norms in one, several, or all components of language (WHO 2022). While the "essential (required) diagnostic requirements" for DLD in the ICD-11 specify that language deficits are "not better accounted for by Autism Spectrum Disorder," the "Boundary with Autism Spectrum Disorder" paragraph of the differential diagnosis section concludes that skills underlying the capacity to use language for instrumental purposes should be coded with the functional language impairment qualifier for ASD rather than a separate DLD diagnosis, but that an additional DLD diagnosis should be assigned when there are "additional specific impairments in semantic, syntactic and phonological development" (WHO 2022).

The implementation of these recommendations, in research or clinical settings, is far from obvious, given current understanding of language impairment in ASD. Some researchers have argued that ASD and DLD constitute points on a continuum of the same disorder instead of separate conditions, on the basis of evidenced overlaps in the patterns of language impairment in the two populations (e.g., Bishop 2010). Others consider DLD as a potential comorbid condition to ASD (e.g., Tager-Flusberg 2015). Arguments against concurrent diagnoses point out differences in the linguistic profiles of the population with DLD and ASD, though some conclude that the concept of ASD/DLD comorbidity is altogether misleading: the crux of the matter being that some children with ASD just have poor language skills, likely due to the effect of cognitive, social, and behavioral difficulties on language development (Tomblin 2011).

Understanding the underlying causes of various types of language difficulty is crucial for tailoring precise and effective support, training and coaching. Assessing relevant linguistic domains guided by linguistic theory and relying on linguistically informed tasks is of primary importance in designing effective support. Effective language assessment; however, may be complicated by the presence of weaknesses or strengths in other cognitive domains that interact with language skills, such as Theory of Mind, executive functions, including working memory, more general non-verbal reasoning skills, or statistical learning, resulting in patterns of ‘peaks and valleys’ in the language and cognitive profiles of individuals with ASD. Furthermore, although some researchers connect (some of) autistic individuals’ language difficulties to the first dimension of ASD (qualitative and significant impairment in socio-communicative abilities and social interaction), the second dimension (restrictive, repetitive, and inflexible behavioral patterns, activities or interests) is rarely mentioned as a potential underlying cause of ASD language difficulties. This warrants further research. In summary, the nature and directionality of the interactions between linguistic skills, extralinguistic abilities and ASD characteristics is the subject of current debate.

The aim of this review is to discuss the key role of language in understanding the challenges in general functioning of individuals with ASD. In doing so, we highlight the heterogeneity of linguistic profiles in autistic individuals, in terms of which language components may be impaired and how, and we point to areas of extralinguistic cognition which may be sources of difficulty or, conversely, which may provide extraordinary language-building resources to these people. These considerations, intimately tied to how these skills can be adequately assessed in ASD, across the range of profiles, are the necessary building blocks for meaningful progress regarding the veritable nature of language difficulties in ASD: a comorbid condition, a likely consequence of ASD, or indeed, in a dimensional approach to neurodevelopmental disorders, just one of a host of separate properties which can combine in individuals in different constellations.

## Language and language domains

Language is a key ability that supports and expresses human thought and enables us to communicate, a resource we use to share our thoughts, emotions, and desires, to participate in social processes, and to learn. Language is a system, with a specific architecture and organization; it also encompasses how this system is used for communication. Over the last 65 years (since Chomsky 1957), linguistics has progressed immensely. It has built theoretical models about the architecture of the system of language. Based on these theoretical models, it has described a large number of languages across the globe, and it has also been able to describe how these language systems are used across different groups of people (see Labov 1972). From a developmental perspective, it has described how children acquire language, what the milestones are in language development, and it has developed theoretical models that explain the process of language acquisition (Guasti 2002). Importantly, apart from language development in neurotypically developing children, a considerable amount of research has investigated how language unfolds in children who develop language in an atypical way, including in autism.

A fundamental outcome of the scientific study of language is that language is a complex and multidimensional system that consists of several distinct domains and subdomains:

(i) *Lexicon* (vocabulary): storage of words and their properties (mental dictionary)
(ii) *Structural language*:
  - Phonology: organization of the sound system via segmental units (individual sounds: vowels (V), e.g., *a, o, i*; consonants (C), e.g., *s, t, r*), syllables (ba-by: CV-CV), and prosodic units (e.g., flat tone in *You ate* vs. rising tone in *You ate?*)
  - Morphology: organization of meaningful elements to form words (e.g., verb stem + 3.^rd^ person singular: *walk-s*)
  - Syntax: organization of words into sentences (e.g., *Kim ate an apple* vs. *What did Kim eat?*)
  - Compositional Semantics: derivation of meaning from the structure of words, sentences, and larger units (e.g., *Kim’s mother washed her* (*her* can refer to Kim) vs. *Kim washed her* (*her* cannot refer to Kim))
(iii) *Pragmatics*: use of language in context, integrating aspects of the linguistic and non-linguistic contexts, often rendering implicit meaning (e.g., *Some mammals can swim* → *Not all mammals can swim*)

Each of these domains is complex and entails in each language a large number of abstract rules and patterns that also result from the interaction between these domains and that describe the complex system of language at a high degree of granularity.

There is abundant evidence from studying children and adults who have impaired language (whatever its source–developmental, acquired, childhood social isolation, etc.) that domains of language can be affected differently. Cases of selective impairment, where deficits in one language area occur in a context of other language domains which are intact, provide unequivocal evidence that the system of language comprises distinct domains or subsystems (Curtiss 2013).

Arriving at informative and reliable language profiles for autistic individuals entails study of the different domains of language. No single language score can accurately profile language in autistic individuals. Exclusive recourse to a vocabulary measure to stand for "language," a common practice in studies on ASD, is particularly risky. Lexical knowledge is part of language, but language is not lexical knowledge. To increase our understanding of issues such as the frequency of co-occurrence with language impairment in ASD, and indeed what it means to speak of comorbid language impairment, it is essential to target specific language domains at a high degree of granularity through appropriate tools.

## Language profiles in ASD

There is consensus in the literature that virtually all autistic people exhibit pragmatic abilities that diverge from those of neurotypicals, although this does not apply to all aspects of pragmatics, nor does it concern the same pragmatic abilities in each individual with ASD (see Section "Pragmatic impairment"). Lexical knowledge has been claimed to be a relative strength in some autistic individuals, unrelated to other linguistic domains (Kwok et al. 2015; Mottron 2004; Sukenik and Tuller 2021; Walenski et al. 2006). Apart from pragmatic divergence and possible lexical strength across the board, the following three main language profiles in ASD have been established:

(i) ASD-LN^2^ ('normal language'): Intact structural language (see (ii) in Section "Language and language domains" above) skills, on a par with neurotypical language
(ii) ASD-LI ('language impairment'): Impairment in structural language skills, i.e., phonology and/or morphosyntax
(iii) MV: Minimal verbal abilities: expression limited to a very restricted set of words and short phrases or absence of spoken language.

The following sections discuss these three broad profiles. Section "Pragmatic impairment" concerns ASD-LN, exhibiting pragmatic impairment only, Section "Structural language impairment" ASD-LI, structural language impairment, and Section "Minimal language" MV, minimal language.^3^

### Pragmatic impairment

Since diagnosis for ASD includes, notably, impairments in social communication and social interaction, it is no surprise that *pragmatics*, broadly definable as the use of language in context, is compromised in the majority of autistic people, even in those individuals who display structural language skills within the typical range (Baron-Cohen 1988; Dewey and Everard 1974; Tager-Flusberg 1981; Tager-Flusberg et al. 2005; Young et al. 2005).

In recent years, however, several scholars have highlighted the non-uniformity of pragmatic abilities in ASD. In some cases, good structural language competence appears to be sufficient to derive certain pragmatic (often non-literal, or figurative) meanings of utterances from their literal meaning. Some researchers refer to this part of pragmatics as ‘Linguistic Pragmatics’ (Andrés-Roqueta and Katsos 2017). Others propose that ‘ego-centrically anchored pragmatic phenomena’ do not cause difficulties for autistic people (Kissine 2016). This has been argued for (conventional) indirect speech acts (i.e., knowing that “*Can you* pass me the salt?” counts as a request even if it has the form of a yes/no question, Kissine et al. 2015; Marocchini et al. 2022), for the derivation of scalar implicatures (i.e., enriching “*Some* students failed” to “Not all the students failed”, Chevallier et al. 2010; Su and Su 2015; Hochstein et al. 2018; Pijnacker et al. 2009; but see Pastor-Cerezuela et al. 2018; Schaeken et al. 2018 and Mazzaggio et al. 2021 for different results), and for the processing of presuppositions (i.e., understanding that if “Leo is eating *again*”, he has eaten before, Domaneschi and Bambini 2020).

As for metaphor, another pragmatic phenomenon, there is currently no agreement in the literature concerning the extent to which metaphor interpretation (e.g., understanding that a very warm room can be described as “an oven”) is difficult for autistic individuals with good structural language skills. Since the seminal work of Happé (1993), several scholars have found a delay of metaphor acquisition in the ASD population. However, Norbury (2005) alleged that this delay could be related to language delay in general, and not to autism per se (see also Kalandadze et al. 2018): difficulties in figurative speech were found to be marginal or even non-significant in subgroups with neurotypical structural language. Yet other authors have found differences in metaphor comprehension in autism even when the autistic and the comparison groups are matched on structural language (Chahboun et al. 2016). These mixed results may relate to the type of tasks that are used (Kalandadze et al. 2019), to the type of metaphors (Mazzarella and Noveck 2021), or to the absence or presence of a literal option in the design (Vicente and Falkum 2021). As for other types of figurative language, such as idioms (a phrase having a figurative meaning not straightforwardly derivable from the meaning of its parts—e.g., *kick the bucket* ‘die’) and proverbs (a sentence which expresses traditional wisdom—e.g., *all that glitters is not gold*) (Morsanyi and Stamenkovic 2021), and metonymies (figure of speech which refers to something by naming something that is closely related—e.g., *the crown* to refer to a monarch) (Melogno et al. 2012), a widespread impairment is found.

Although difficulties with pragmatics may relate to impairments in structural language (see Section "Structural language impairment"), they could also directly stem from autistic traits, such as weak central coherence (Vulchanova et al. 2015), strict rule-following (Vicente and Falkum 2021), and, in particular, from a deficit in Theory of Mind (ToM) (for elaboration on ToM, see Section “Theory of Mind (ToM)”). ToM (or mindreading, mentalizing, perspective-taking) is the ability to reason about one’s own and others’ mental states, including beliefs, intentions, desires, and emotions, and to understand and predict behavior based on these (Premack and Woodruff 1978).^4^ Certain pragmatic phenomena involve the social context and the perspective of others, for example of the individuals involved in the conversation. Such pragmatics requires ToM (Andrés-Roqueta and Katsos 2017; Kissine 2016, 2021; Mognon et al. 2021) and has been coined ‘Social Pragmatics’ by Andrés-Roqueta and Katsos (2017). Social pragmatics includes, among other things, irony, some cases of metonymy, reference in conversation, and storytelling (narratives). For example, the comprehension of irony is viewed by almost all scholars as an ability that necessarily calls for mentalistic skills, because when a speaker has said something that is clearly false (e.g., “What a wonderful day” when it is raining), interlocutors have to reason about what the speaker knows about their own knowledge to distinguish lies from irony (Sullivan et al. 1995). Since ASD is characterized by difficulties in the areas of ToM (Baron-Cohen 1990; Baron-Cohen et al. 1985; Peterson et al. 2012), and in keeping track of diverse perspectives (Kissine 2012), it is not surprising that many studies have found a deficit in irony comprehension (Deliens et al. 2018; Happé 1993; Kaland et al. 2002; MacKay and Shaw 2004; Martin and McDonald 2004; Saban-Bezalel et al. 2019). In recent years, however, other studies have not found a delay in irony comprehension in autistic children, at least at the behavioral level, even if the latter may process irony differently than neurotypical children in real time, as shown by implicit measures (response times and eye gaze, Pexman et al. 2011) or brain imaging techniques (Colich et al. 2012; Wang et al. 2006; Williams et al. 2013a, b). This fact might suggest that, whereas many autistic individuals stick to the literal interpretation of sentences and thus misunderstand irony (Vicente and Falkum 2021), others may find an alternative, albeit more cumbersome, route to solve the problem posed by irony, mediated by their verbal abilities (see also Panzeri et al. 2022).

Mindreading is also required for the interpretation and use of referential expressions (Durrleman and Delage 2016; Kuijper et al. 2015; Marinis et al. 2013; Novogrodsky and Edelson 2016; Overweg et al. 2018). The choice between explicit and more informative descriptions (e.g., “the nurse”) and potentially ambiguous personal pronouns (e.g., “she”), for instance, is guided by their pragmatic relevance in a specific context, to avoid redundancy on the one hand and ambiguity on the other. Autistic individuals experience problems both in comprehension and production of referential expressions. These problems are not limited to spoken language but have also been found in signing autistic children (see Shield et al. 2015). Autistic individuals have been shown to have difficulty using pronouns appropriately in the specific context, including first and second person pronouns, i.e. *I, me, you*, leading to cases of pronoun reversal (Naigles et al. 2016; Overweg et al. 2018), and using nonambiguous and adequately informative referential expressions (Novogrodsky and Edelson 2016; but see Stegenwallner-Schütz and Adani 2020). In null-subject languages such as Italian and Greek (Mazzaggio and Shield 2020; Terzi et al. 2019), instead of using ambiguous pronouns, autistic children tend to avoid pronouns in favor of nouns or names. These difficulties have been found to inhibit the listener’s comprehension of a story (Marinis et al. 2013). They have been attested in ASD in different languages with different grammars, for example in Dutch, a language that encodes definiteness vs. indefiniteness (Schaeffer et al. 2018) as well as in Mandarin, which has no morphological marker of definiteness (Sah 2018). The attested difficulties do not seem to depend on linguistic abilities solely (e.g., morphosyntactic skills; Meir and Novogrodsky 2023), suggesting that also in this case mindreading abilities are involved, to consider the perspective of the conversational partner, or to keep track of the common ground shared by speaker and hearer (knowledge that is common to conversational partners).

Competent narrative discourse, or storytelling, which involves describing a series of events that are contingent on one another, can also involve taking into account other people’s mental states. Concerning narrative abilities, mixed results have been found for the ability to use language appropriately, for example to tell a coherent story. While some studies found no differences between autistic children and their typically developing peers in their narrative abilities (e.g., Capps et al. 2000; Kuijper et al. 2015), other studies found that autistic children, besides using more ambiguous pronouns (Novogrodsky 2013; Suh et al. 2014; Peristeri et al. 2020), use less mental state language (Baron-Cohen et al. 1986; Brown et al. 2012; Peristeri et al. 2017) and fewer causal conjunctions such as *because* (Losh and Capps 2003), compared to typically developing children, and that these difficulties may continue into adulthood (Geelhand et al. 2020). In addition, children with ASD tend to tell stories using linear, coordinated (versus hierarchical) chains of events as a core event structure of their stories (Peristeri et al. 2017). These findings suggest that pragmatic limitations cannot be overcome by good language in a contextualized task like narratives (Peristeri et al. 2017). Weak central coherence, often argued to be impaired in individuals with ASD, can also affect their narratives (production and comprehension), as it causes interpreting utterances in isolation rather than integrating information from many sources (Norbury and Bishop 2002).

Note that the mixed results do not seem to be entirely explainable from differences in the need for mindreading and weak central coherence (but see Kuijper et al. 2015), and hence seem to reflect other differences between the studies such as the specific tasks used and the populations tested. In particular, as the consideration of other people’s mental states seems relevant for particular aspects of narrative production, but not for others, this may explain why children with ASD do not show pragmatic difficulties across the board.

To summarize, pragmatic phenomena representing an interface between language and mindreading (Social Pragmatics) emerge as impaired in ASD, showing both receptive and expressive difficulties. When linguistic competence is sufficient for re-interpreting words, statements and discourse (Linguistic Pragmatics), autistic people without structural language impairment may show typical performance and abilities, at least on the surface.

### Structural language impairment

As mentioned in Section "Language and language domains", structural aspects of language include sound structure (phonology/phonetics), word structure (morphology), phrase and sentence structure (syntax), and phrase and sentence meaning (semantics). As presented in Section "Language and language domains" as well, based on structural language skills, two main profiles in autistic individuals have been reported: ASD-LN (‘language normal’) and ASD-LI (‘language impairment’). Currently, a major question regarding the latter profile is to what extent some of these individuals may have low performance for reasons other than their structural language abilities. In other words, is ASD-LI part of ASD or is ASD-LI comorbidity of ASD and DLD (Developmental Language Disorder)? (see Bishop 2010; Bishop et al. 2016; Tager-Flusberg 2015; Rice 2016). Intimately entwined in this debate are the questions of which autistic individuals are included in studies reporting on language abilities and which types of tools are used to assess these abilities.

#### Prevalence of structural language impairment in verbal autism

While structural language impairment is clearly not universal in autism, there is no accepted prevalence estimate that emerges from current research. Studies such as Loucas et al. (2008) or Kjelgaard and Tager-Flusberg (2001) are frequently cited to indicate that roughly half of verbal autistic children display structural language impairment. Loucas et al. (2008) conclude that 57% of verbal children with ASD (41/72 participants in their study) display language impairment; all of these children have NVIQ (Non-verbal IQ)^5^ scores of ≥ 80, and LI is defined as a score ≤ 77 on Receptive Language, Expressive Language or Total scores on the CELF-3 (Clinical Evaluation of language Fundamentals—Semel et al. 2000). Kjelgaard and Tager-Flusberg’s (2001) study of 89 autistic children, which includes children with the full range of IQ scores, report CELF summary language scores below 70 for 48% (21 of the 44 children who were able to complete the tasks). These two studies illustrate the two major difficulties in determining structural language impairment prevalence in (verbal) ASD: population bias (exclusion of children with both extremely low, < 70, and low IQ, 70–79, and sometimes even children in the low average range) and low task completion rates, which are generally indicative of difficulty with task demands, interference due to poor pragmatic, reasoning, and other extralinguistic cognitive skills, even for many children who did complete the tasks, making interpretation of scores extremely difficult (Tager-Flusberg 2000a, b; Kjelgaard and Tager-Flusberg 2001).

Regarding tools used for language assessment in ASD, the challenge posed by the need for autism-appropriate tasks has been taken up by the LACA network (https://laca.humanities.uva.nl/wp/), which recommends a specific type of production task, repetition, for baseline measures of structural aspects of language. Repetition tasks involve very limited task demands: instructions are maximally simple, they do not involve inferencing from pictures or previous linguistic material, and memory load and the influence of lexical knowledge can be minimized. Moreover, there is ample evidence that nonword repetition (NWR), for phonology, and sentence repetition (SR), for morphosyntax, are (the most) reliable way to screen for structural language impairment, including independently of autism (Armon-Lotem and Meir 2016; Chiat 2015; Conti-Ramsden et al. 2001; Fleckstein et al. 2018; Marinis and Armon-Lotem 2015; Rujas et al. 2021; a.o.) and constitute reliable measures of phonological and morphosyntactic skills, respectively.

#### Phonology and prosody

While some studies suggest that only autistic individuals with intellectual disability (ID) have a phonological deficit (Boucher 2003), others have clearly shown the presence of such a deficit, even in individuals without ID (Bishop et al. 2004; Demouy et al. 2011; McCleery et al. 2006; Rapin et al. 2009).^6^ Kjelgaard and Tager-Flusberg (2001) found that, in general, articulatory skills are relatively unaffected in children with ASD, but that a subset of children show deficits in their ability to repeat nonwords, without mentioning deficits in phonological skills. In contrast, Rapin et al. (2009) explicitly identified 24% in a group of children with ASD aged 7–9 years who demonstrate severe and persistent phonological impairment, which has been related to immature syllabic development (Paul et al. 2011) or to a chronological shift in phonemic^7^ development (Wolk and Edwards 1993; Wolk and Giesen 2000; Wolk et al. 2016).

Phonological analysis of spontaneous language samples from autistic children reveals interesting observations. This method allowed Wolk and colleagues to highlight atypical patterns of phonological acquisition alongside a delay in phonological development (McCleery et al. 2006). Atypical patterns were also observed by Wu et al. (2020), particularly in complex syllable structures (e.g., syllables including consonant clusters as in the English word *strict*). These atypicalities were observed in very young children with ASD (Schoen et al. 2011; Sheinkopf et al. 2000), including in studies restricted to children without ID (Cleland et al. 2010). Paul et al. (2011) identify fewer vocalizations, fewer consonants, and fewer consonant types in very young children at high risk for autism whose general developmental indices were within norms. At the syllable level, these at-risk children produced fewer syllables (including canonical consonant + vowel syllables, i.e., CV syllables) and fewer syllable types at nine months of age.

The major difficulty in interpreting findings investigating sound structure in autism based on production tasks is that the results typically depend directly on how performance is tested and analyzed. For example, Kjelgaard and Tager-Flusberg (2001) concluded that standardized tests do not allow for fine-grained testing of structures that may cause difficulties, or for distinguishing processing or task management difficulties from underlying language deficits. Indeed, their 2001 study and that of Whitehouse et al. (2008) used the NEPSY pseudoword repetition task (Korkman et al. 1998), which is constructed to test short-term memory. It contains 13 items of different lengths (2–5 syllables) that are close to real words in English, and phonological complexity is not controlled for (e.g., 'bwelextiss’). Thus, poor performance on this task may not be indicative of impaired phonology. Rather, it may be due to deficits in memory, articulation or lexicon, or a combination of several of these factors. In fact, nonword repetition tasks are generally based on the number of syllables, and thus low scores are attributed to memory limitations (Bishop et al. 2004; Riches et al. 2011). In contrast to this practice, Silleresi et al. (2020) used a NWR task based on phonological complexity that keeps syllable length to a minimum and that contains nonword that are not word-like in the target language (dos Santos and Ferré 2018). They found that, similarly to children with DLD, some children with ASD had weak performance, showing that clear *phonological* difficulties, sometimes severe, can be observed in ASD.

We now turn to prosody, an area that is often reported to be unusual in autistic language. Prosody serves essential communication functions at the social/pragmatic level and includes accentual facts, rhythm and intonation. These are expressed by measurable acoustic correlates such as variations in duration, intensity and pitch. These acoustic parameters combine in complex ways across languages. Prosodic complexity also lies in the fact that prosody has several functions: it can be extralinguistic in that it identifies characteristics of the speaker (gender, age, dialect), paralinguistic because it conveys emotion, and linguistic in that it signals pragmatic aspects such as the communication situation and the speaker's state of mind (irony, sarcasm, neutrality), but also structural language aspects: prosody participates in the realization and structuration of the different linguistic levels (including lexicon, syntax, semantics and pragmatics) (see also McCann and Peppé 2003, for a distinction of different types of prosody).

Prosody in the language of autistic people is often described as unusual or even deviant, seldom used to enhance communication (e.g. Bishop et al. 2004; Green and Tobin 2009; McCann and Peppé 2003; Nadig and Shaw 2012; Shriberg et al. 2001). While there is consensus that emotional and pragmatic aspects of prosody are difficult for autistic individuals, some studies have also reported poorer performance in the processing of structural language aspects of prosody, in both production and comprehension, such as lexical stress, intonation, or phrase boundaries (Paul et al. 2005; Peppé et al. 2011; Shriberg et al. 2001).

Regarding perception, adolescents with autism have been shown to have poorer performance than their neurotypical peers in lexical accent processing (Paul et al. 2005). In Lyons et al. (2014), poor performance was restricted to autistic individuals with LI. Unsurprisingly, due to their social communication difficulties, children with autism struggle with contrastive stress (when focus is placed on what distinguishes two referents*, it’s the RED car, not the black one*) regardless of their NVIQ scores (Peppé et al. 2011). Autistic children without ID were found to show more errors in phrasing (how pauses are distributed within a sentence) than neurotypical children (Lyons et al. 2014; Shriberg et al. 2001). Furthermore, autistic children with a structural language impairment were found to perform more poorly than neurotypical children on a comprehension task testing prosodic cues (level intonation vs. rising intonation) to determine (non-)interrogative readings of certain words in otherwise identical sentences (Huang et al. 2021). In contrast, no perceptual differences in chunking (the prosodic delimitation of word grouping, e.g., *cream # cheese # and yogurt* vs. *cream cheese # and yogurt*) have been observed in children with autism (Fine et al. 1991; Paul et al. 2005).

On the production side, pitch has been widely investigated, but there is no consensus regarding the indices that can effectively characterize the prosody of autistic individuals. Some studies found higher pitch measures than in neurotypical children (Chen et al. 2021; Olivati et al. 2017; van Santen et al. 2010; a.o.), while others reported results equivalent to those of neurotypical children (Dahlgren et al. 2018; Diehl and Paul 2013; Hubbard and Trauner 2007; Paul et al. 2008; a.o.).

Mixed results also found in studies measuring intensity or duration and flow. Hubbard and Trauner (2007) observed the range of vowel intensity (in repetition and spontaneous speech) and found significant variation between participants with and without autism. The same pattern was found for the intensity of elicited utterances (minimum, maximum, and range intensity) by Olivati et al. (2017), whereas no difference on a mean intensity measure was reported by van Santen et al. (2010) using the PEPS-C (Peppé and McCann 2003). Likewise, no consensus emerges from studies examining duration or flow rate (a.o. Arciuli et al. 2020; Dahlgren et al. 2018; Diehl and Paul 2013; Hubbard and Trauner 2007, Kissine and Geelhand 2019; Olivati et al 2017; Paul et al. 2008; van Santen et al. 2010).

The general lack of consensus as to whether and which aspects of prosody are impaired in ASD may originate from the widely varying methodologies, but also from the inherent variability of the subjects themselves: with or without ID, children, adolescents or adults, with structural language impairment or not. Furthermore, as was also mentioned in Section "Pragmatic impairment" on pragmatics, surface similarities with neurotypicals may still reflect different underlying processing. This is underscored by Eigsti et al.’s (2012) MRI study revealing different regions of activation between autistic and neurotypical adolescents when they listened to statements and questions that were contrasted by prosody only. The authors found a more generalized activation of neural regions in autistic than in neurotypical participants, suggesting that autistic individuals make broader use of executive brain areas. This is interpreted as less automatic language processing by individuals with ASD. Finally, as also suggested by Diehl et al (2015), tools used to assess prosody must be as ‘pure’/specific and controlled as possible, so as not to bring in other language or cognitive dimensions that could lead to erroneous conclusions.

#### Morphosyntax

A bulk of work investigating morphosyntax (which refers to the ordinary usage of the word *grammar–*syntax and the morphological properties of words related to their position in a sentence) in autistic children has brought forth evidence showing that some children show intact performance on par with their neurotypical peers, while other autistic children present difficulties comprehending and producing a variety of morphosyntactic structures (e.g., Al-Hasan and Marinis 2021; Arutiunian et al. 2021; Durrleman and Delage 2016; Kjelgaard and Tager-Flusberg 2001; Meir and Novogrodsky 2020; Modyanova et al. 2017; Perovic et al. 2013; Riches et al. 2010; Schaeffer 2018, 2021; Silleresi et al. 2020; Tager-Flusberg 2006; Terzi et al. 2014; Williams et al. 2013a, b). Affected morphosyntactic structures are those, notably, whose computation has been argued to be complex in that it entails syntactic dependencies called syntactic movement in which a constituent is pronounced in a different position from that in which it is interpreted (e.g., *Which child did the mother hug?* where *which child* is pronounced at the beginning of the sentence, but interpreted as the patient of the verb *hug*, canonically expressed in the position following this word) and/or entails an embedded clause (e.g. *We know the child [that the mother hugged]*).

From investigation into the loci of morphosyntactic difficulties and the resemblance of structural language profiles of children with ASD to those of children with DLD, two major findings have emerged. Some studies have found that morphosyntactic language profiles of children with ASD-LI only partially resemble those of children with DLD. For example, Durrleman et al. (2017), who tested various types of passives vs. active sentences in ASD, found that children with ASD performed poorly on passive sentences, but many of them also performed poorly on active sentences unlike what is typically found for children with DLD. This ASD/DLD difference could have been a result of the fact that comprehension was ascertained via a choice among four pictures, which requires skills often weak in autism (Naigles and Fein 2017). In the same vein, Sukenik and Friedmann (2018) showed that participants with ASD exhibited poor performance across various syntactic structures including simple as well as syntactically complex ones. This latter study explicitly noted, moreover, that error analysis showed that reduced performance may be affected by pragmatic impairment and not necessarily caused by pure morphosyntactic difficulty, unlike what is observed in children with DLD. The study by Huang et al. (2021) discussed above on the interaction between prosodic and morphosyntactic skills suggests something similar. These results suggest that at least some reported morphosyntactic difficulties could be intimately related to autism and autistic symptomatology, notably pragmatic and/or prosodic difficulties.

A number of studies have nonetheless reported important overlaps in the morphosyntactic profiles of children with ASD and those with DLD, suggesting comorbidity of ASD and DLD in some children. For example, Meir and Novogrodsky (2020) found that children with ASD-LI aged 4;6–9;2 showed impaired skills similar to those reported in the literature for children with DLD and were particularly challenged with structures including syntactic movement and/or multiple clauses. Error pattern analysis showed that the children with ASD-LI simplified complex structures (see also Riches et al. 2010), a pattern also observed in children with DLD. See also Prévost et al. 2018, on the production of object and subject pronouns (clitics) in French, in which children with ASD displayed a remarkably similar pattern of intact and impaired pronouns to that of children with DLD.

It is still an open question whether autistic individuals with structural language impairment display difficulties with all types of structural language, including phonology, phonetics, morphology, syntax and compositional semantics. One study that addresses this question is Tuller et al. (2023). They analyzed the lexical, phonological and syntactic skills of 51 autistic children and found every possible combination of impaired and spared language skills; notably, each of these domains could be spared or impaired independently of the two others (see also Rapin et al. 2009). This provides evidence for the dissociation of the relevant language domains (see Section "Language and language domains"), but also raises questions regarding potential comorbidity of structural language impairment and ASD. If structural language impairment is a consequence of ASD characteristics (and thus not a condition comorbid with ASD), then why would not all types of structural language always be impaired? One possible answer is that individual variation in ASD characteristics yield selective structural language impairment. Yet, the results of the study by Tuller et al. (2023) resemble those found for DLD, which also displays variations in types of language impairment (cf. Friedmann and Novogrodsky 2008).

### Minimal language

Language emergence tends to be delayed in ASD (see Section "Language Trajectories"). Although most autistic children ultimately become verbal, a significant proportion, about 30% of school-age children and autistic adults, remain Minimally Verbal (MV), meaning that they have only a few words or phrases, or lack “functional” verbal language (Howlin et al. 2014; Kasari et al. 2013; Norrelgen et al. 2015; Pickles et al. 2014). It must be pointed out immediately that reported prevalences of MV children may vary greatly as a function of measures used and criteria (Bal et al. 2016). This shows how crucial it is for research papers to explicitly specify how these children are identified (see Posar and Visconti 2022, for an overview).

Although some children may develop alternative ways of communicating (e.g., via pointing, gesturing, imitating or pictograms/pictures), the question arises as to the nature of the linguistic competence of MV children. Are there different (receptive) language profiles in MV children? Are they entirely without a structural language system, or do they have some receptive language abilities, and if so, do these abilities differ from those of neurotypical children and those of verbal autistic children? Another question concerns intellectual development in MV children. Do these children tend to have intellectual deficiency, or can they develop typical intellectual functioning? Also, do MV children tend to have more severe autism symptoms than verbal children? It is striking that although the prevalence of MV children is far from being non-negligible, they nonetheless remain an under-researched population. Yet, families and clinicians are in crucial need of answers concerning language prognosis for these children and how to better communicate with them.

Assessing language abilities of MV children through standardized tests commonly used in speech-language therapy, including receptive language tasks, can be very challenging, as these tasks often contain complex instructions and require active participant responses. Results may thus not reflect these children’s real language capacities. Instead, it is recommended that language skills of MV children be investigated via tools generating automatic, passive responses, notably eye-tracking and EEG (Brady et al. 2014; Tager-Flusberg et al. 2017), although these are not without their own difficulties. For instance, despite elaborate guidelines meant to gradually make MV participants get used to wearing a scalp EEG cap, Tager-Flusberg et al. (2017) report a 40% completion rate for their EEG protocols in children and 70% in adolescents.The (few) studies that have implemented such techniques have mainly focused on the meaning of words, reporting atypical responses in MV children. In two recent studies, ERP responses were measured while MV and neurotypical children were performing a word-picture matching task. Responses to visual stimuli were found to be delayed and have lower amplitude in the MV children, suggesting impaired visual processing, which in turn may have a negative impact on vocabulary acquisition (Ortiz-Mantilla et al. 2019). No N400 effect was found in the MV children in the mismatch condition, in contrast to the neurotypicals (Cantiani et al. 2016). These results seem to go in the same direction as the findings of a brain anatomy study showing that the volume of the left planum temporale, which is associated with lexical processing, was smaller in autistic individuals with LI than in those without LI (Knaus et al. 2018). However, variability in the findings reported in lexical semantics studies is high. In Cantiani et al. (2016), a N400 effect was detected in half of the MV participants, suggesting that MV children may not be a monolithic population, with some children displaying lexical semantic processing similar to neurotypicals and others having atypical processing mechanisms. Results from a word learning study go in the same direction: some MV children were able to learn and retain new words, while others did not (Joseph et al. 2019).

With respect to structural aspects of language, evidence for comprehension of basic word order has been found. Studies using the Intermodal Preferential Looking paradigm have shown that a number of young children, despite having extremely limited expressive skills, looked preferably at a matching video when hearing a stimulus sentence (with basic Subject-Verb-Object-SVO-word order), compared to a mismatching video (Swensen et al. 2007; Su and Naigles 2019). Using a scaffolding learning technique, whereby children were first trained on practice sentences (in English), Schneider and Hopp (2011) found that MV individuals could perform correctly in a (SVO) sentence-picture matching task. These studies, although limited, suggest that MV children are able to develop morphosyntactic rules, in contrast to the interpretation developed in Slušná et al. (2019), according to which MV children have no internal grammar. Work on structural language abilities in MV children should clearly be complemented by investigation of the comprehension and processing of noncanonical word order phenomena, which involve varying degrees of morphosyntactic complexity (see Section “Phonology and prosody”), as well as phonological phenomena, which have not yet been studied in this population.

Turning to extralinguistic cognition, MV children are often assumed to be cognitively impaired (Bal et al. 2016). However, some MV children do develop typical or borderline NVIQ (see also Section  “Non-verbal IQ (NVIQ)”) and/or other extralinguistic abilities. Concerning autism severity, measures used in studies comparing MV and verbal individuals with ASD (e.g., the ADOS Calibrated Severity Score, social affect score and Restricted and Repetitive Behaviors [RRB] score, Lord et al. 2012), including individuals with language impairment, generally indicate that MV children do not score significantly higher than verbal individuals, which would otherwise be indicative of more severe symptoms (Knaus et al. 2018; Plesa Skwerer et al. 2019; Thurm et al. 2015). Comparing raw total scores for social affect and RRB across the three ADOS modules, however, Hus et al. (2014) noted that individuals assessed via module 1 (so, with less language) tended to score higher than more verbally fluent individuals, particularly those assessed via Module 3, although no statistical analysis was presented. It is yet to be determined whether language performance on tests specifically designed for MV individuals is predicted by autism symptom severity.

## Language trajectories

As emphasized repeatedly throughout this article, language capacities in ASD are very heterogeneous, not only in terms of general “functional” capacities, but also according to the extent to which specific language domains may be affected or preserved, the so-called peaks and valleys depiction. Even each broad language profile as defined in Section "Language profiles in ASD" covers much heterogeneity. Although autistic people in the ASD-LN profile may have preserved structural language abilities, they vary considerably in their pragmatic skills. Similarly, autistic people in the ASD-LI profile show varying pragmatic skills, and, besides that, may not all exhibit fragility in the same linguistic domains and to the same extent. Furthermore, MV autistic people may vary with respect to how much language they produce: some do not speak at all, others produce some spoken language but the numbers of spoken words and phrases may vary per person. Moreover, absence or minimal presence of spoken language can coincide with strengths in language comprehension, or with relatively good reading and writing skills. To date, there is no detailed picture of the various language profiles occurring within the three broad profiles defined in Section "Language profiles in ASD". Furthermore, whether the linguistic heterogeneity found among autistic individuals is due to associated cognitive, neurodevelopmental or even genetic factors is far from clear. The LACA network has taken upon itself to further investigate these two questions: detailed descriptions of autistic language profiles and understanding their underlying mechanisms. Moreover, LACA finds it equally important to be able to determine how autistic people’s language capacities develop with age. In particular, is it possible to identify and predict developmental trajectories regarding language in autism, during childhood and throughout the lifespan? Answers to the questions posed above should make it possible to provide the most appropriate remediation and support. The current section discusses what we know about language trajectories in autistic individuals to date.

We first note that language emergence is delayed in many autistic children. While in neurotypical development first words and first word combinations (phrases or sentences) are found at around 12 months and 24 months respectively, in autism these milestones can come significantly later. In a retrospective study of early development in 162 autistic children, 70% did not have phrases at age 33 months (Grandgeorge et al. 2009). It is not clear at this point why language emergence is slower in some autistic children than in others. One potential explanation draws upon children’s impaired interactional skills and lack of interest in others (Whitehouse et al. 2007), which is compatible with an approach to language development in which communication is the driving force underlying language acquisition (Goldberg 2013; Tomasello 2008). In particular, joint attention has been proposed to be a significant predictor of language development in autism (see Bottema-Beutel, 2016 for an over-view). While joint attention may indeed play an important role in the very early phases of language acquisition, it appears to be much less predictive of language outcome (Kissine 2021). Moreover, there is evidence that joint attention may not be impaired from birth on, but rather follows a progressive decline from age 2 to 6 months (Jones and Klin 2013). If this is the case, then delayed language emergence may not be directly linked to joint attention deficiency. One possibility is that some autistic children may show slow language growth as a result of difficulties integrating speech sounds and mouth movements (Kissine et al. 2021), as part of early atypical sensory perception, in all modalities, which may lead to later atypical cognitive profiles (Bonnet-Brilhault et al. 2017).

Thus, while joint attention may facilitate language acquisition, it appears not crucial, as is also evidenced by the fact that many autistic children (around 70%) acquire fluent language (Kissine 2021). This is further underscored by reports of autistic children who acquire second languages through watching TV, without interaction, and so without joint attention (Kissine et al. 2019; Vulchanova et al. 2012). The question is then how autistic children do acquire language, if not through joint attention and social interaction. One proposal is that autistic children rely more heavily on so-called statistical learning (Kissine 2021). Statistical learning is the ability to effortlessly detect patterns and regularities in (auditory, visual or visuo-motor) input without explicit instruction or intention to do so. It is hypothesized to support the acquisition of language by allowing children to identify and internalize the complex structures of natural languages. It has been shown to be related to language processing in adults (Daltrozzo et al. 2017; Misyak et al. 2010) and children (Kidd and Arciuli 2016) and to be impaired in DLD (Lammertink et al. 2020). It is easy to imagine that statistical learning is supported by strong systemizing skills (*hyper-systemizing*), which autistic individuals are often argued to have as a possible instantiation of a second dimension characteristic: sensory hyper-sensitivity (Baron-Cohen et al. 2009). One fascinating example of how such skills support language learning in ASD is the autistic savant Christopher, who has very strong systemizing skills and has learned to speak, understand and write more than twenty languages (Smith and Tsimpli 1995).

A meta-analysis by Obeid et al. (2016) suggests that while language disorders like DLD may be associated with a significant deficit in statistical learning, no such deficit is confirmed for ASD. However, the findings of this study should be taken with caution: ASD is a wide spectrum, and the studies included in this meta-analysis often exhibit small and heterogeneous samples of participants, without taking into consideration individual differences in IQ, severity of autism symptoms or language abilities. Furthermore, other studies that are either more recent than Obeid et al.’s review, or are neuroimaging instead of behavioral studies, suggest that despite neurotypical statistical learning outcomes in ASD, more sensitive methodology can identify differences in the underlying processing strategies and learning trajectories of autistic individuals (Jeste et al. 2015; Jones et al. 2018; Scott-Van Zeeland et al. 2010; Zwart et al. 2017; 2018). Recall that the hypothesis of diverging processing strategies in ASD is entertained for some pragmatic abilities in ASD as well (see Section "Pragmatic impairment"). For autistic adults, some recent evidence has suggested that statistical learning is associated with language abilities (Parks et al. 2020).

A minority of recent studies also found differences in statistical learning outcomes, in the form of an advantage for (visual) statistical learning in autistic learners (Roser et al. 2015), or a correlation between poorer social cognition (in the general population) and better statistical learning (Zwart et al. 2017).

In short, the mixed findings on statistical learning in ASD suggest that broad generalizations are difficult to make and could obscure differences across the spectrum. More research is needed to establish if, how and to what extent the different language profiles and language-developmental trajectories of autistic individuals may be explained by statistical learning abilities.

Investigating whether language outcome in ASD may be predicted by atypical sensory processing is another interesting line of research. Atypical sensory processing of both social (Ben-Sasson et al. 2007) and nonsocial (Williams et al. 2021) stimulations has been reported in the early years, as well as atypical audio-visual integration, as seen above (Kissine et al. 2021). Aberrancies in sensory processing circuits during early development may lead to a cascade of cognitive and social deficits, including atypical neural specialization for speech and processing of speech, which may impede language acquisition and communication skills (Bonnet-Brilhault et al. 2017).

Another characteristics of language development in autism is language regression, or a language plateau, which refer to children who have lost previously acquired skills, or have stopped progressing. According to a recent meta-analysis, language regression begins between 15 and 30 months (mean age 21 months), and its prevalence is about 25%, which may be higher depending on how the population has been sampled (Barger et al. 2013). In their retrospective study of 196 children with any kind of language regression or plateau (the vast majority of which met ASD diagnostic criteria), Wilson et al. (2003) found that language regression was generally severe (70% became nonverbal) and that although some language recovery was possible, full recovery was very rare. Little is known about what causes language regression. Comparing autistic children (mean age 41.4 months) grouped according to their language development trajectories (regression, plateau, general delay, no delay), Jones and Campbell (2010) found no inter-group differences related to autism symptomatology and non-language developmental history. Wilson et al. found that language regression in autism was associated with “more global autism regression”. One outstanding issue is whether regression can affect language domains selectively or whether it concerns all aspects of language at the same time. Tracking language regression precisely and prospectively, with appropriate language tools, would allow for detection of subtle ability loss in autistic children, which has been argued to be under-reported in the literature (Pearson et al. 2018).

Despite frequent slow language emergence as well as frequent language regression, language skills in individuals performing below norms have been found to improve with age. In their meta-analysis of 92 longitudinal studies focusing on language development, Brignell et al. (2018) reported language gains during childhood, albeit not always statistically significant, despite great variability across studies regarding language measures (standardized tools, expressive and receptive vocabulary tests, and parent reports), participant characteristics (e.g., with or without ID), and time elapsed between baseline and follow-up. Although in many studies the rate of progress was comparable (or even faster) to reference or age-equivalent norms, language scores were nonetheless below norms, at both baseline and follow-up. Some studies also reported on verbal and nonverbal children at baseline and outcome. The percentage of verbal children was found to increase by 20% on average, with large discrepancies across studies. Increases in verbal language tended to be larger in children aged 5 and under (19–30%) than in children over age 5 (5–32%). Predictors of language outcome are not very clear. In some studies, it is reported that language level and nonverbal IQ at baseline are predictors of language outcomes at follow-up, but this was not found in Brignell et al.’s meta-study, presumably due to different methodologies and population samples across studies.

In the few studies that have investigated language abilities post early adolescence, there seems to be a slowdown in language gain. Despite general improvement between childhood and adulthood, few autistic adults reach a neurotypical level for their age (see Magiati et al. 2014 for an over-view) and between 10 and 33% have minimal language (Lord et al. 2018; see also Section "Minimal language"). Late adolescence and early adulthood correspond to time periods during which social relationships tend to become increasingly complex, which may be particularly taxing with respect to language use and comprehension, and impact developmental trajectories (Alpern and Zager 2007; Picci and Scherf 2015).^8^

The results reported in longitudinal studies should be interpreted with caution, however, mainly due to methodological concerns. The Brignell et al.’s (2018) meta-analysis reports medium to high risks of bias in 66–93% of the publications they reviewed, based on study participation, study attrition and outcome measures. Moreover, the language data are either indirect (e.g., answers to parental questionnaires) or limited, with one language domain, vocabulary, being targeted much more often than others. When tested, morphosyntax was mainly assessed through omnibus tests and/or tests that are inappropriate for many autistic individuals, such as the CELF (see Section “Prevalence of structural language impairment in verbal autism”). As to phonology, it has largely been ignored, and when it was evaluated, it was assessed through tasks heavily relying on memory capacities.

Finally, how language fares with cognitive aging in autism remains largely unknown. Yet, older autistic adults have their own specific needs which should be addressed in order to improve their quality of life, notably through adapted support and care. A decline in various cognitive functions, such as executive functions, including episodic memory and working memory, and processing speed, has been shown in neurotypicals (Park et al. 2002). These cognitive changes are accompanied by brain reorganization mechanisms, such as additional activations in frontal regions, which could be compensatory (Park and Reuter-Lorenz 2009). Studies of cognitive aging in ASD have reported contradictory results, some suggesting that autism is vulnerable to cognitive aging (Roestorf et al. 2019) and others proposing that ASD acts as a protective factor, including in the case of a neurodegenerative disorder (Oberman and Pascual-Leone 2014). It remains to be seen whether the different language domains are impacted by aging cognitive functions in autism, and how language skills relate to potential patterns of brain reorganization.

In sum, there is a need to document more precisely language-developmental trajectories and language in adulthood in ASD, looking at a wider range of language domains and a wide range of individuals, and using appropriate language tools. For morphosyntax and phonology, this could be achieved, in verbal individuals, by using child and adult versions of specific kinds of sentence repetition and nonword repetition tasks (see also Section "Structural language impairment").^9^ For MV individuals, similar tasks generating passive responses (e.g., eye-tracking and EEG paradigms) could be used at different time points throughout the lifespan, and are currently being developed.

## The influence of extralinguistic cognition on linguistic ability

As mentioned in some of the previous sections, linguistic abilities sometimes interact with extralinguistic (or: nonlinguistic) cognitive functions, such as ToM, Executive Function or intelligence (expressed by IQ). As autistic people’s extralinguistic abilities are often reported to diverge from neurotypicals’, it is important to discuss them in more detail and to consider including them in a broad assessment battery testing linguistic skills. This is exactly what the LACA network attempts to do in the LACA Baseline Battery, which proposes tasks for evaluation of both linguistic skills and nonlinguistic cognitive skills. The current section discusses in more detail ToM, EF and IQ in ASD.

### Theory of Mind (ToM)

As noted in Section "Pragmatic impairment" on pragmatics, ToM (or: mindreading, mentalizing) is the ability to reason about one’s own and others’ mental states, including beliefs, intentions, desires, and emotions, and to understand and predict behavior based on these (Premack and Woodruff 1978). ToM is fundamental for a variety of social interactions, including conversations, coordinating, cooperating, conflict resolutions and gaining popularity amongst peers (Astington 2003; Astington and Edward 2010; Astington and Pelletier 2005; Derksen et al. 2018; Mazza et al. 2017; Sally and Hill 2006). ToM has been argued to be a core impairment in ASD (Baron-Cohen 1990; Baron-Cohen et al. 1985; Yirmiya et al. 1998) which may explain differences in communicative and social skills characteristic of the autistic condition (APA 2013). However, note that Gernsbacher and Yeargeau’s (2019) contest the claim that autistic individuals are uniquely or universally impaired in ToM, based on a review of empirical evidence, failure to replicate original findings, and failure to show associations between ToM results and, for example, autistic traits and social interaction.

Besides the relation between ToM and pragmatics (see Section "Structural language impairment"), other linguistic skills relate to ToM in autistic and neurotypical individuals, in particular, syntax. The ability to attribute beliefs, in particular false beliefs, is an important step in ToM development. Success at false belief tasks, such as the classic Sally-Anne task (Baron-Cohen et al. 1985, adapted from Wimmer and Perner 1983),^10^ has been shown to be closely related to syntactic competency. For instance, syntax emerges as more closely tied to false belief task success than lexical skills in neurotypical children in the meta-analysis by Milligan et al. (2007). In children with autism as well, vocabulary appears to have a lesser impact on false belief task performance than syntax (Fisher et al. 2005; Tager-Flusberg 2000a, b; Tager-Flusberg and Joseph 2005; Tager-Flusberg and Sullivan 1994).

Scholars have also highlighted the role of specific grammatical structures for belief reasoning. For instance, Milligan et al., (2007) revealed that specific mastery of complement clauses, namely sentences of the type: *The toddler believes/thinks/says that Santa Clause exists*, accounts for most of neurotypical children’s variance in false belief understanding, above and beyond that of both general lexical and global syntactic abilities (Astington and Jenkins 1999; de Villiers and Pyers 2002; Durrleman and Franck 2015; Durrleman et al. 2016; Tager-Flusberg and Joseph 2005; although see also Fontana et al. 2018). The interest of complementation for belief reasoning has been articulated in terms of its specific relevance as a cognitive tool for misrepresentation (de Villiers and de Villiers 2000; de Villiers and Pyers 2002). Thus, in the sentence *The toddler believes/thinks/says that Santa Clause exists*, the entire sentence remains true even though the complement (*that Santa Claus exists*) it contains is false (since Santa Clause does not exist). As such, this structure captures an essential property of (subjective) beliefs, namely that they can be in contradiction with (objective) reality. However, another crucial characteristic of complements is that they allow meta-representation, namely the capacity to represent another representation, or hold in mind the content of another mind. Given their specific deficit in ToM, autistic children would be “especially dependent on language, particularly knowledge of sentential complements, to bootstrap their meta-representational capacity” (Tager-Flusberg and Joseph 2005). Indeed, according to a meta-analysis by Farrar et al. (2017), children with developmental delays such as ASD seem to rely more specifically on complementation than their neurotypical peers in the realm of ToM understanding. In line with the idea that complementation sentences allow the representation of beliefs (de Villiers 2007, 2021), training studies targeting the comprehension of complements have also given rise to higher performance on false belief tasks in both neurotypical on the verge of consolidating ToM (Hale and Tager-Flusberg 2003; Lohmann and Tomasello 2003; Shuliang et al. 2014) and older children with ASD (Durrleman et al. 2019, 2022).

Studies on neurotypical children (Pérez-Leroux 1998; Smith et al. 2003) as well as on those with ASD (Durrleman et al. 2018) have found that mastery of relative clauses (e.g., *The toddler that kicked Santa; The child who wanted gifts)* by preschool neurotypical children also relates to false-belief task performance, although these do not allow misrepresentation. Still, as training on relative clauses in neurotypical children has been reported to fail at boosting false belief task success, in contrast to training on complements (Hale and Tager-Flusberg 2003), it could be that structures that allow both meta-representation and misrepresentation (hence complements) are those that are indeed mostly tightly entwined with successful mentalizing.

### Executive function (EF)

Executive functions (EF) refer to higher-order cognitive processes involved in goal-directed behavior, such as inhibition, shifting, planning or updating in working memory. Working memory refers to the ability to store verbal or nonverbal information while performing a cognitive task. Quantitative and/or qualitative impairment in EF, which may persist even when general intelligence is controlled for, has been reported in individuals with ASD (Joseph et al. 2005; Merchán-Naranjo et al. 2016; Robinson et al. 2009; a.o.). An executive function account of autism suggesting that social and non-social difficulties in individuals with autism are due to their EF deficits has even been proposed (see Hill 2004, for a review). However, this hypothesis has been undermined as it cannot account for all the symptoms in autism and as significant interindividual variability in EF performance has been reported in this population (Liss et al. 2001; Pellicano 2010; Ellis Weismer et al. 2018). Thus, EF impairment that is reported at a group level is not necessarily observed at an individual level, suggesting that EF impairment is not a core deficit in individuals with ASD (Friedman and Sterling 2019). Moreover, studies have so far reached inconsistent results concerning EF impairment in ASD, including incongruent results obtained for the same EF construct. These intra-study and between-study divergences may be related to factors such as the characteristics of the participants with ASD (e.g. age, cognitive level, autism symptomatology/severity, language ability), the characteristics of the control groups (e.g. Typically Developing, ADHD, DLD) or the type of EF tasks used (e.g. verbal/non-verbal; level of complexity, purity) (Chen et al. 2016; Ellis Weismer et al. 2018; Friedman and Sterling 2019;). These interindividual and intergroup differences are partly a reflection of the heterogeneity of cognitive profiles in autism and of the developmental change that may occur during the lifespan in this population.

Language and EF skills are argued to be linked in typical development (see Ellis Weismer et al. 2018, for a review), which poses the question of whether a similar link between these abilities exists in individuals with ASD. While associations between EF and structural language measures in children with ASD have been reported in several studies, the nature and the directionality of this relationship remain unclear. Measures of EF (e.g., shifting, working memory) are shown to predict performance on language tasks such as word meaning and morphosyntactic processing tasks (Ellis Weismer et al. 2017; Haebig et al. 2015) in children with ASD and training of working memory is reported to be associated with improvement in receptive and expressive syntax in this population (Delage et al. 2021). On the other hand, receptive and expressive language measures are reported to predict performance on EF (e.g., shifting, inhibition, updating in working memory) in children with ASD—with or without language impairment—similarly to neurotypical children matched on age (Ellis Weismer et al. 2018). Some authors (Edmunds et al. 2022; Liss et al. 2001) suggest that performance of children with ASD on EF tasks is mediated by language via inner-speech (self-talk). Others (Joseph et al. 2005) argue that these children have a deficit in the use of language for verbal self-regulation while performing EF tasks. However, these researchers propose that this deficit is independent of the children’s language skills as no significant correlation between EF tasks (working memory, inhibitory, and planning) and language measures is found in the ASD group, even after controlling for non-verbal ability.

Associations between EF and pragmatic measures have also been reported (for a review, see Friedman and Sterling 2019). Results for this association are, once again, mixed, as some studies have shown that EF predict/mediate pragmatic abilities in individuals with ASD (Filipe et al. 2019) while others did not (Cardillo et al. 2021). Similarly, although some authors suggest that impairment in skills underlying social pragmatic performance in individuals with ASD, such as perspective taking, are related to EF limitations like dysfunction in cognitive flexibility (Kissine 2012), others do not reach the same conclusion (Deliens et al. 2018). According to a longitudinal study by Akbar et al. (2013), language skills predict (verbal) working memory (but not organization, inhibition and shifting) performance when both structural language and pragmatic abilities are taken into account.

In sum, to date, research does not give a clear picture about EF deficits and the nature of their relationship to language in individuals with ASD. Most of these studies are cross-sectional, limited to group analyses and involve autistic individuals without ID. Further studies exploring group, subgroup (e.g. language normal (LN) *vs.* language impaired (LI)) and individual data and including individuals with autism on the entire spectrum, with or without language impairment and with variable cognitive abilities and age ranges, may be necessary to better catch the link between language and EF variability. Finally, longitudinal studies may be useful to elucidate the directionality of the relationship between language and EF, knowing that better understanding of this relationship in individuals with ASD may have direct clinical implications. Although the nature of the relationship between language and EF/working memory in individuals with ASD is still unclear, the assessment of individuals with ASD must include indices of EF and working memory, in addition to language measures, to obtain a full picture of one’s strengths and weaknesses and to develop a tailor-made intervention for each individual with ASD.

### Non-verbal IQ (NVIQ)

Intelligence can be measured through language-based reasoning or via nonlinguistic means. If we want to investigate the relationship between language and intelligence, and if language can be impaired in ASD, it seems only logical to use measures of IQ that do not rely on using language in autistic individuals (to be elaborated on at the end of this section). Of all autistic people, two-thirds have neurotypical intelligence; this leaves one-third of autistic individuals with ID (Maenner et al. 2020).

In the past decades, interest among researchers (Georgiades et al. 2013; Harris 2019; Lai et al. 2013; Lombardo et al. 2019; a.o.) and clinicians (DSM-5, APA 2013 and ICD-11, WHO 2022) in delineating different subgroups within the ASD population has grown. To date, several studies have taken up the question of the nature and heterogeneity of intelligence profiles (Audras-Torrent et al. 2021; Feczko et al. 2018; a.o.) independent of language abilities. From a clinical perspective, the ICD-11 (WHO 2022) identifies and describes five different profiles in ASD. Four of these profiles belong to the verbal part of the spectrum. They derive from all logically possible combinations of either spared or impaired functional language and intellectual abilities. Adapting the ICD-11 to classification to impaired structural language, this entails two “homogeneous” profiles, ASD-LN without ID and ASD-LI with ID, and two “discrepant” profiles, ASD-LI without ID and ASD-LN with ID. A fifth profile concerns absence of functional language, which is found in MV children with ASD (see Section "Minimal language"), combined with ID. In addition to what is reported in the ICD-11, a few studies have evoked the existence of a sixth profile characterizing MV children without ID (16% MV children in Bal et al. 2016).

The possible existence of these profiles, and in particular those showing a discrepancy between linguistic and intellectual abilities, raises several theoretical considerations in the domain of research on language abilities in autism. First, it undermines the traditional assumption that spared intellectual abilities necessarily lead to spared language abilities, and that impaired intellectual abilities entail *ipso facto* impaired structural language skills. Moreover, from the perspective that language domains can be selectively spared or impaired (see Section "Language and language domains") (even though this domain interfaces nonetheless with other modules and central systems) these results receive a natural interpretation. Adapting the words of Smith and Tsimpli (1995), the existence of these varied profiles provides a classical example of *double dissociation*: language can be impaired or even absent in some children on the autism spectrum with otherwise intact intellectual abilities, and—more surprisingly—some children on the autism spectrum with ID may nonetheless have intact, or even enhanced, linguistic ability. In the literature regarding formal language abilities in verbal children with autism, there are very few studies that have found evidence for all four profiles of verbal children in their population samples (Joseph et al. 2002; Kjelgaard and Tager-Flusberg 2001; Miniscalco and Carlsson 2021; Silleresi et al. 2020; Tuller et al. 2017). Two possible methodological stumbling blocks may be responsible for this, which question – with direct support from experimental data—the assumption that language is an automatic consequence of intellectual abilities in autism. The first one is linked to the paucity of studies that have included verbal children with intellectual impairment in their population samples. This relative infrequency (see Silleresi 2018) means that there is a lack of knowledge about the language capabilities of these individuals, especially those corresponding to the ASD-LN with impaired intelligence profile. In this vein, Tuller et al. (2017) highlight that a real understanding of linguistic/intelligence profiles in autism can only be achieved through investigation of the entire spectrum, which is not restricted to children without ID (corresponding to only 70% of the spectrum). The second methodological weakness is related to how intellectual abilities are evaluated in studies on language in autism. For one thing, the psychometric tools employed across studies include different indices and different subtests targeting different abilities (verbal, nonverbal, full-scale IQ, working memory, etc.). Such differences make it impossible to compare profiles of abilities from one study to another. In addition, many studies use IQ tests that rely on language abilities, which leads to circularity if put into relation with language measures (Silleresi et al. 2020).

## Summary and perspectives

This review focused on how language functions and develops in autism, with the aim of setting the stage for a discussion of what the nature of language impairment in ASD may contribute to a dimensional approach to neurodevelopmental disorders. We emphasized that language, in autism and more generally, is not monolithic, but rather comprises different components, corresponding to different abilities. Language in autism is indeed often cited as a *prima facie* case of selective language impairment because the domain of pragmatics is taken to be impaired across autistic individuals, even in those with typical language abilities in other language domains. In contrast, lexical knowledge has been claimed to be a relative strength in some autistic individuals, despite potential impairment in other language domains. However, as has become evident throughout this article, these generalizations do not hold across all autistic individuals—there is large individual variability. A fundamental implication is that no single language measure can adequately assess language abilities in autism.

We addressed questions regarding ASD language profiles with varying degrees of (selective) impairment and questions with respect to potential comorbidity of autism and language impairment. Three broad ASD language profiles were identified, namely, (i) minimally verbal autistic individuals, (ii) verbal autistic individuals without structural language impairment, and (iii) verbal autistic individuals with structural language impairment. Each of these three groups includes various sub-profiles.

Irrespective of the tripartite distinction sketched above, one consensus in the field is that all autistic individuals have difficulty with pragmatics, although selective impairment within pragmatics seems to occur as well. As discussed in Section "Pragmatic impairment", not all parts of pragmatics are impaired, and not all autistic people have difficulty with the same pragmatic phenomena. Yet, pragmatic phenomena that require mindreading, or ToM (such as metaphor, irony, presupposition) cause difficulty in most autistic people, often leading to misunderstandings or communication break-downs.

In addition to pragmatic difficulties, a sizeable proportion of autistic individuals have difficulties with structural language (notably, phonology and morphosyntax), as noted above under (iii). Striking similarities with the structural language profile found in DLD have been reported for many verbal autistic individuals, in particular, children. At the same time, quantitative and qualitative structural language differences from DLD have also been observed. One subdomain of structural language is underinvestigated, namely, compositional semantics (the derivation of meaning from morphosyntactic structure), warranting future research.

Some other areas of language require more research to establish their relative strengths or weaknesses in ASD. Prosody, a domain which is often reported to be unusual or deviant in people with ASD, is mostly known for emotional prosody, whereas the few studies on structural linguistic prosody and pragmatic prosody provide mixed results. Furthermore, although a few studies report lexical skills to be strong in some autistic individuals, more studies are needed in this area.

Regarding language assessment in ASD, the following observations were spotlighted. Many language tasks included in routinely used standardized language batteries involve complex verbal instructions and rely on multiple extralinguistic cognitive abilities, including reasoning and inferencing skills, working memory, ability to integrate details from pictures into a coherent mental construction, etc. Many of these extralinguistic cognitive skills are areas of known weakness for many/most autistic individuals. As a result, if individuals show low performance in language tasks, it is unclear whether this reflects deficits in specific language domains or whether it results from deficits in extralinguistic cognitive abilities. In turn, extralinguistic cognition (including ToM, EF, IQ) is often tested through verbal tasks, making it difficult to disentangle it from language abilities. If autistic individuals score poorly on tests that assess extralinguistic abilities through language, it is the question whether this low performance stems from weakness in extralinguistic cognition or from language difficulty. Furthermore, characteristics related to the second dimension of ASD (restrictive, repetitive, and inflexible behavioral patterns, activities or interests), such as perseverance, inflexibility, sensorial sensitivity and systemizing may negatively or positively affect language development and language processing, and therefore language task performance. Finally, appropriate and adequate baseline tools to assess certain areas of language in ASD, such as pragmatic, prosodic and compositional semantic abilities, are virtually non-existent. The same applies to tools assessing the linguistic skills of minimally verbal autistic individuals.

The way forward to disentangling language profiles in autism requires carefully designed studies, taking into account (a) the respective language domains, (b) the relevant aspects of extralinguistic cognition and autism severity, and c) the influence of linguistic difficulties on extralinguistic cognitive performance. Each test in a language assessment battery should be as ‘pure’ and specific as possible. For example, structural language assessments must control for pragmatics and other linguistic (e.g., lexicon) and extralinguistic (e.g., ToM) cognitive domains which can influence structural language performance in autistic individuals. Pragmatic assessment should focus on pragmatic phenomena that seem particularly vulnerable in ASD, namely, the ones that require mindreading or perspective-taking, and that cannot be tackled by structural language or lexical skills alone. Prosody assessment must distinguish between emotional, pragmatic and structural language prosody. Furthermore, language assessment batteries need to include tests on extralinguistic cognition such as ToM, EF, IQ, central coherence, statistical learning, and on autism severity, including second dimension characteristics, such as perseverance, inflexibility, sensorial sensitivity, systemizing. Moreover, such skills should be assessed non-verbally, so that they are not obscured by impaired language. Finally, strong efforts should be made to assess the language skills of minimally verbal autistic individuals, for example, by developing special tools testing language comprehension without requiring active responses.

A start toward this fundamental, methodological goal has been made by the LACA network, which is developing the so-called “LACA Baseline Battery”. The aim is to have screening tools for all linguistic and relevant extralinguistic domains, including autism severity. Good progress has been made for the structural language area, in particular, for morphosyntax and phonology. Ideas for adequate and appropriate ToM and pragmatics tests are currently being developed. A challenge to be overcome in the LACA Baseline Battery is the goal to have appropriate assessment tools across the life-span, so for toddlers, school-age children, adolescents, adults and the elderly.

Turning now to the issue of comorbidity, a lively, ongoing debate has emerged about whether ASD can be comorbid with DLD. Some studies treat the structural language difficulty condition as a co-occurring condition, e.g., in the ASD-LI profile. The few studies that exist on this topic usually compare ASD-LI groups to ASD-LN groups on the one hand, and to DLD groups on the other, to understand what might be specific about LI in ASD. However, such studies often do not simultaneously compare properties related to ASD in the two ASD groups and their link (or lack thereof) with structural language impairment in each group. There is no model of how structural language difficulties may interact with ASD characteristics and with various extralinguistic cognitive abilities. This currently prevents us from taking a firm position in the debate as to whether DLD can be comorbid with ASD or regarding the question as to whether ASD and co-occurring language difficulty would be better approached from a dimensional view to neurodevelopmental disorders. As discussed in Section "Structural language impairment", some recent studies reveal subtle differences in terms of structural language error patterns and quantities between ASD-LI and DLD. These studies suggest that, even at a phenotype level, the structural language difficulties in ASD are not identical to those encountered in DLD. If these results get replicated, and moreover, if research shows that structural language difficulties in ASD do not have the same underlying causes as DLD, this suggests absence of DLD as a possible comorbid condition to ASD. Such a position would be further strengthened by observations of language-developmental trajectories in autistic children that are different from those of children with DLD or neurotypical children. A potential future conclusion that DLD as it is currently defined (see Section "Introduction") is not comorbid with ASD would fit a dimensional view to neurodevelopmental disorders, namely, that ASD hosts a variety of properties (including potential language impairments) which can combine in individuals in different constellations.

The questions raised by the considerations and observations in the paragraph above are: “Are particular structural language difficulties in ASD direct consequences of certain ASD characteristics (including the second dimension)? Or do (certain) ASD-characteristics and co-occurring structural language difficulties follow from a third underlying cause?” It is these questions that we recommend investigating in future research, in parallel to describing the various autistic language profiles in detail. This can be achieved only if the various linguistic and relevant extralinguistic skills are assessed by means of tests that are as ‘pure’ and specific to the relevant area as possible. This will allow us to uncover fine-grained potential associations between various linguistic abilities, extralinguistic cognitive abilities and autistic traits. Moreover, it will further enable us to provide scientific input to the development of urgently needed, more appropriate, precise and individualized support and training for both autistic individuals and their non-autistic communication partners.

Concluding, there is little research on how characteristics usually associated with ASD interact with co-occurring language difficulties. The current tripartite distinction between minimally verbal autism, ASD-LI and ASD-LN hides an enormous linguistic heterogeneity. A good part of this heterogeneity may be explained if more attention is paid to the way linguistic and extralinguistic cognitive abilities and autistic traits are assessed. Only when more detailed language profiles in ASD have been described can we start answering questions regarding potential comorbidity of ASD and DLD or whether co-occurring language impairment needs to be approached from a dimensional view to neurodevelopmental disorders.

## Data Availability

Data sharing is not applicable to this article as no original datasets were generated or analysed during the current study.

## Footnotes

1 The appellation *Autism Spectrum Disorder (ASD)* is the term used by the current versions of the WHO's International Classification of Diseases (ICD-11, WHO 2019/2021), the international health data standard, and the widely used Diagnostic and Statistical Manual of Mental Disorders (DSM-5) of the American Psychiatric Association (APA 2013). We therefore use this term, while acknowledging that some prefer the term *condition* in place of *disorder*. To refer to people diagnosed with ASD, acknowledging the ongoing controversy within the relevant communities, *autistic people*, *people with ASD*, *people with autism*, etc. are used interchangeably. Finally, the term language impairment (LI) or language disorder is used to refer to significant, ongoing language difficulties, without regard to their origin, acknowledging that some prefer the terms *difficulties* or *needs* instead of *impairment* or *disorder*.

2 The terms ASD-LN and ASD-LI are based on the terms ALN (Autism Language Normal) and ALI (Autism Language Impaired), first introduced by Tager-Flusberg (2006).

3 The issue of echolalia is not discussed in this paper. Although echolalia is commonly assumed to be a distinctive characteristic of autism, Gernsbacher et al. (2016) point out that there is no empirical support for this claim. Their review of echolalia, pronoun reversal, and production-comprehension lags in autism clearly shows that none of these is quantitatively or qualitatively exclusive to autism or to neurodevelopmental disorder. Echolalia can constitute a stepping stone to more productive language use, even though it may be a disrupting factor in social interaction (pragmatics). As our review focuses on pragmatic and structural language abilities, and echolalia does not appear to tell us much about these abilities in autistic individuals, we decided not to include echolalia in our discussion.

4 We follow the current state of the literature in using several related terms: *mentalizing*, the general ability to reason in terms of mental states, *Theory of Mind* or *mindreading,* the ability to understand that others have intentions, desires, beliefs, perceptions, and emotions different from one’s own and that such intentions, desires, and so forth affect people’s actions and behaviors, and *mindblindness*, the inability to understand others’ behavior in terms of belief–desire reasoning (see APA dictionary of psychology https://dictionary.apa.org).

5 Section “Non-verbal IQ (NVIQ)” adresses the question of which IQ measures are relevant for studies of language in ASD.

6 Most of the studies on phonology in autism are conducted on children.

7 A phoneme is the smallest unit of speech that distinguishes one word from another. For example, the sound *p* in *tap*, distinguishes that word from *tab*, *tag*, and *tan*. Therefore, *p* is a phoneme in English.

8 It should also be noted that some individuals only receive a diagnosis of ASD in adulthood. In many cases, this corresponds to individuals with good language skills and an IQ within norms (whose compensatory mechanisms are presumably overrun by the demands of adulthood), or/and individuals whose autistic characteristics had been misdiagnosed in childhood (see Mandy et al. 2018). As is the case with autistic adults with an early diagnosis, detailed investigation of the language abilities of those individuals has largely been ignored.

9 LITMUS tasks (Language Impairment Testing in Multilingual Settings, https://www.bi-sli.org/litmus-tools) are recommended by the LACA network (Language Abilities in Children with Autism, https://laca.humanities.uva.nl/wp/.

10 In the Sally-Anne task, the participant needs to understand that Sally falsely believes that a marble is in the basket since she did not observe Anne moving the marble to the box (although the participant did observe this, and thus knows what the truth is).
